# Supplementary material for: A Comprehensive Assessment of Ultraviolet-Radiation-Induced Mutations in Flammulina filiformis Using Whole-Genome Resequencing
Source: J Fungi (Basel). 2024 Mar 20;10(3):228. doi: 10.3390/jof10030228 (PMC10971301; doi:10.3390/jof10030228)
Supplement: Supplementary file 1 [file jof-10-00228-s001.zip › Supplementary Material S8/KEGG annotation/out/64381550635650.os/KO/out_map/map04391.html]

KEGG PATHWAY: Hippo signaling pathway - fly - Reference pathway


|  |  |
| --- | --- |
| **Hippo signaling pathway - fly - Reference pathway** |  |

[
Pathway menu
| Organism menu
| Pathway entry
| Show description
| User data mapping
]

Hippo signaling is an evolutionarily conserved signaling pathway that controls organ size during development from flies to humans. The core of the drosophila pathway is a kinase cascade consisting of four proteins including Hippo, Salvador, Warts and Mats. Upon activation by stimuli such as high cell density in cell culture, the Hippo pathway kinase cascade phosphorylates and inhibits the transcriptional co-activator Yorkie, thereby promoting cell apoptosis and restricting organ size overgrowth. When the Hippo pathway is inactivated, Yorkie translocates into the nucleus to bind to transcriptional factors to promote cell growth and proliferation.

|  |  |
| --- | --- |
| Reference pathway | 100% |
